# Erlotinib therapy for Olmsted syndrome with p.L655P missense mutation in the *TRPV3* gene: a case report

**DOI:** 10.3389/fmed.2025.1512673

**Published:** 2025-01-24

**Authors:** Jia Zhang, MengYue Guo, DongYang Yuan, JinYang Wei, Hongzhou Cui

**Affiliations:** ^1^Department of Dermatology, Changzhi People's Hospital, Changzhi, China; ^2^Department of Dermatology, First Hospital of Shanxi Medical University, Taiyuan, China

**Keywords:** Olmsted syndrome, erlotinib, *TRPV3* gene, missense mutation, case report

## Abstract

Olmsted syndrome (OS) is a rare disorder characterized by a mutilating palmoplantar keratoderma and periorificial keratotic plaques, but which shows considerable clinical heterogeneity. Recently, transient receptor potential vanilloid 3 (*TRPV3*) mutations associated with autosomal dominant or recessive OS have been reported. Here we describe a classically OS case with definitive diagnosis of OS based on clinical features and a genetic assay. Genetic analysis revealed heterozygous variants in the *TRPV3* gene using whole-exome sequencing of case-parents’ trios. This mutation was not identified in his mother. Notably, a previously unreported heterozygous frameshift mutation, c.1964 T > C (p.L655P), was identified in exon 15 of the *TRPV3* gene in this patient and his father. Additionally, the patient was effectively managed with oral erlotinib at a daily dose of 75 mg. After 3 months of treatment, most plantar lesions resolved, and the pain experienced was mildly alleviated. No significant adverse effects were observed in this case during treatment. In addition, we review the OS literature regarding *TRPV3* gene mutations.

## Introduction

Olmsted Syndrome (OS) is a rare genodermatosis classically characterized bilateral progressive mutilating palmoplanter keratoderma (PPK) and periorificial keratotic plaque that gradually progress over time (1). This syndrome was first described in 1927 by Olmsted (2). The disease usually begins at birth or in early childhood, and the clinical presentation is quite heterogeneous. The most suggestive symptoms associate PPK with pseudoainhum and periorificial keratotic plaques (3, 4). Uncommon cutaneous manifestations such as nail dystrophy, leukokeratosis of the oral mucosa, and hyperhidrosis or hypohidrosis of the palmoplantar region may also be observed in association with OS (5). OS affects both genders, with a higher prevalence in males. Genetic testing provides the most reliable method for accurately diagnosing OS, although only a limited number of cases with gene mutations have been identified since its initial detection. Whole-exome sequencing has revealed pathogenic mutations in transient receptor potential vanilloid 3 (*TRPV3*), a gene responsible for encoding a particular type of ion channel, in individuals with autosomal-dominant OS (6, 7). Mutations in PERP (TP53 apoptosis effector) have also been reported to cause OS (8). Cheong et al. performed comparative exome sequencing and Sanger sequencing to identify a G-to-A transition at position c.573 in the *TRPV3* gene, producing the missense mutation p.Gly573Ser in the proband (9). Subsequent studies have found a series of mutation sites, including p.Leu673Phe (10), p.Trp692Cys (11), p.Gly573Cys (12), and p.Gly568Val (13). In recent years, an increasing number of mutation sites have been identified, indicating the genetic heterogeneity of OS inheritance. Notably, Zhong et al. identified five previously unreported variations of the *TRPV3* gene: *R416Q*, *R416W*, *L655P*, *W692S*, and *L694P* (14). Of particular interest is the L673F variant, which has been associated with olfactory sensory (OS) dysfunction without the presence of periorificial keratoderma, thereby categorizing it as an atypical form of OS. Present therapeutic interventions for OS are frequently inadequate and may provide temporary symptomatic relief. In a study by Greco et al., three patients with OS exhibited resolution of *TRPV3* mutation-associated PPK with erlotinib hydrochloride treatment (15). In 2022, Kathleen et al. reported an another case of erlotinib-induced remission of pain and PPK in a patient with *TRPV3* mutation-associated OS (16). However, erlotinib-induced remission of PPK in the Chinese patient with *TRPV3* mutation-associated OS has not been reported yet.

In this study, we report a case of OS in which patients present with PPK and skin peeling on the extremities. Initially, the characteristic lesion of the proband’s plantar was examined histopathologically and ultrastructurally. Additionally, we screened *TRPV3*, which was previously indicated in the pathogenesis of genodermatosis with mutilating palmoplantar keratoderma. The patient’s symptoms were effectively improved by oral erlotinib at a daily dose of 75 mg. To our knowledge, this is the first report of a Chinese patient with OS harboring a missense p.L655P mutation in *TRPV3*.

## Case report

**Figure 1:**
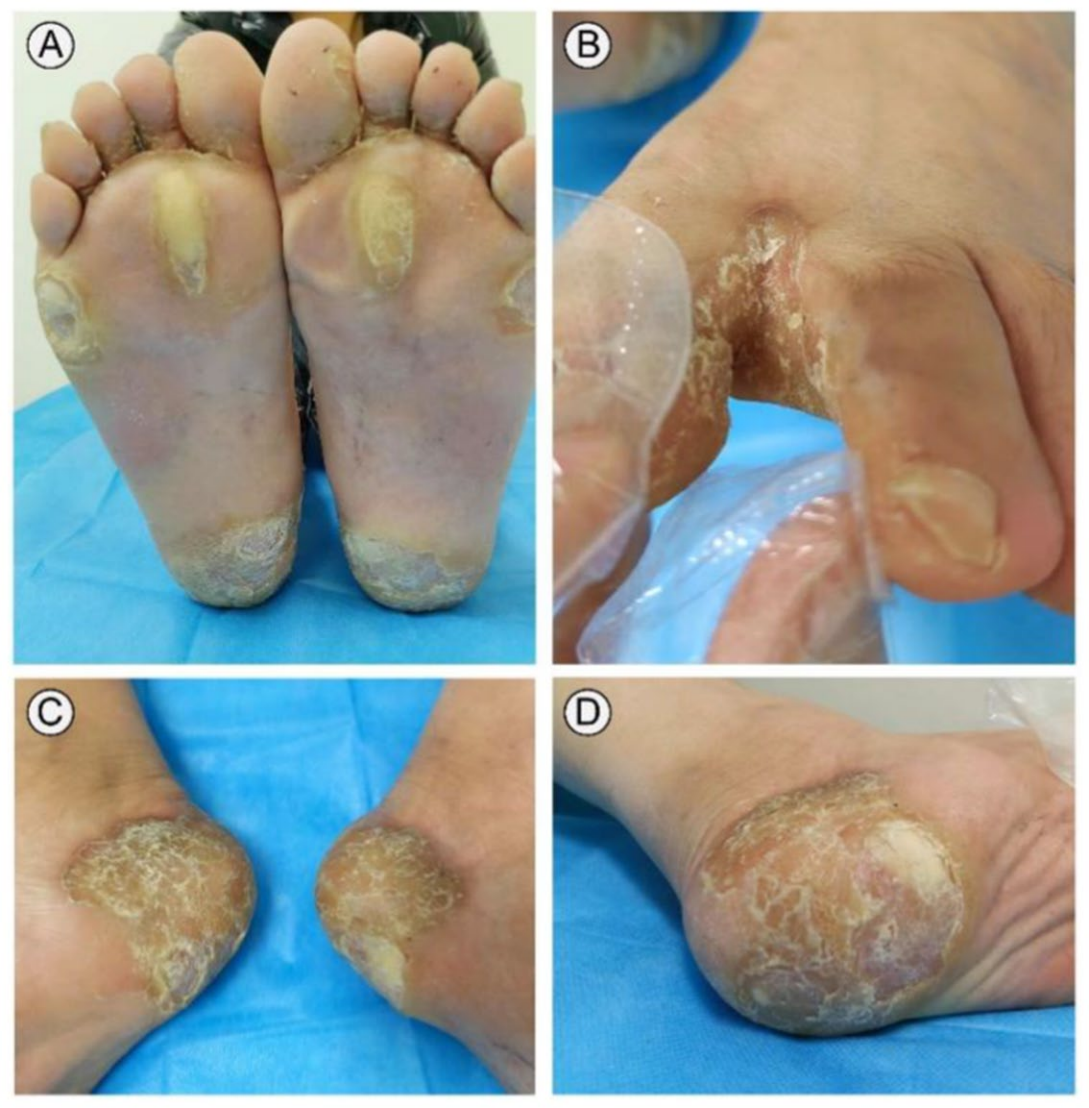
Clinical presentation of the case. The patient presented with symmetric, focal palmoplantar keratoderma and obvious pseudoainhum on the weight-bearing areas of both feet **(A,C)**. Palm region keratinous scale was observed in toe interdigital, desquamation and warmth in the extremities **(B,D)**.

**Figure 2:**
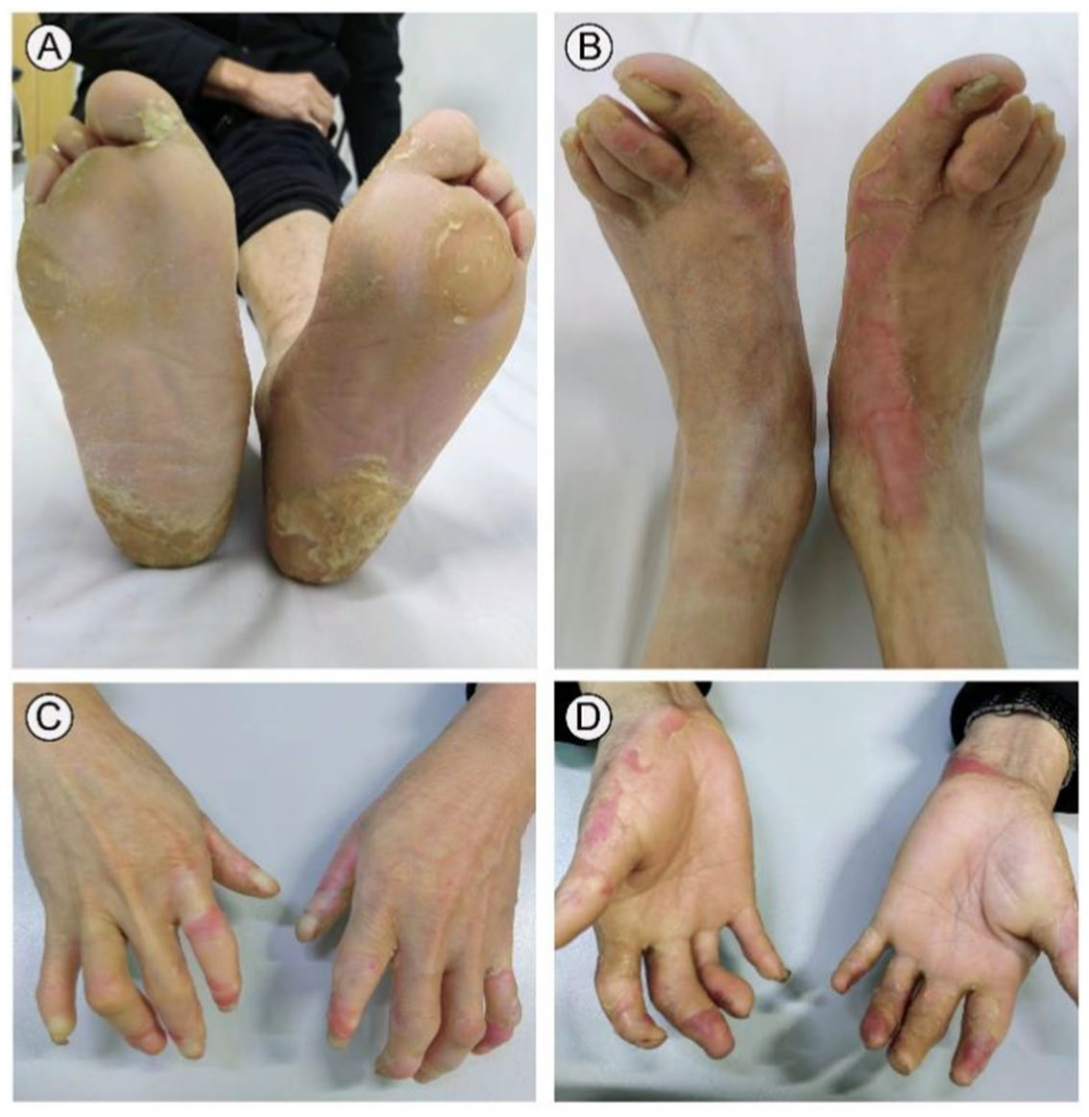
Clinical presentation of the patient’s father. His father yellow showed thickened and dry hard, hypertrophic keratotic patches, clear erythema on both feet **(A,B)**. Cone-shaped fingers and desquamation on the palms were observed on both hands **(C,D)**.

A 31-year-old male presented to our outpatient clinic with symptoms including symmetrical focal PPK, intermittent episodes of warmth-induced pain and itching, sclerodactyly-like features, mild pseudoainhum, and skin peeling on the extremities (Figures 1A,B). The patient’s illness lasted for about 8 years. He presented with mild keratosis and peeling, and thick yellow-brown, fissure hyperkeratic plaques on the pressure sites of the plantar (Figures 1C,D). The hyperkeratotic patches were focal in nature, predominantly localized in patches at pressure points, without spreading beyond these areas. The patient suffered from recurrent episodes of painful plaques on his feet. Each episode was marked by intense and debilitating pain that did not improve with various pain medications. The severity of the pain prevented the patient from walking and engaging in sports activities. There were no signs of hyperkeratosis around the mouth or abnormalities in hair and nail structure. Systemic examination did not reveal any abnormalities. His father manifested with focal keratotic plaques, cone-shaped fingers, and desquamation on the palms (Figures 2A–D). Intriguingly, the symptoms of his father were much milder than those in the case. However, symptoms such as: acral hyperalgesia, severe itching, or photophobia, were absent in the two patients. The parents were allegedly nonconsanguineous.

**Figure 3:**
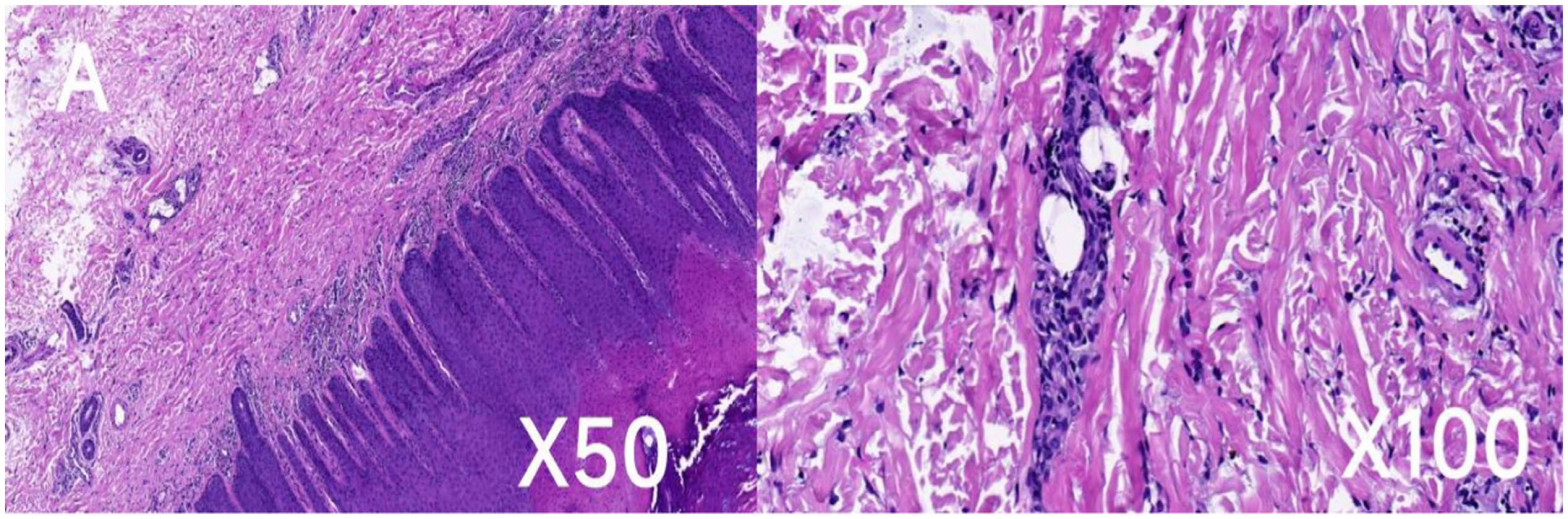
Histopathologic and ultrastructural features of the patient’s skin lesion. Hematoxylin and eosin (H&E) staining of the skin lesion revealed psoriasiform hyperplasia characterized by dense hyperkeratosis **(A)**, thickening of the skin layers (acanthosis), notable parakeratosis in the upper skin layers, reduced granular layer (hypogranulosis), and vesicular degeneration **(B)**. Scale bar: A, ×50; B, ×100.

**Figure 4:**
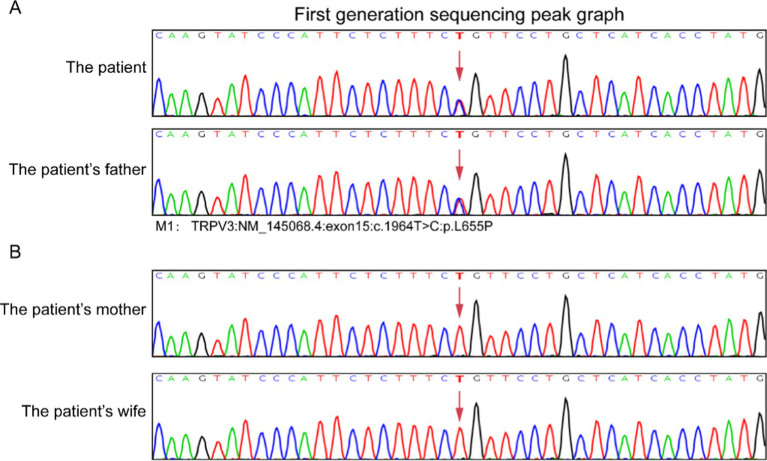
Result of DNA sequencing of *TRPV3* mutation in the patients’ family. *TRPV3* gene sequencing results revealed a identical heterozygous mutation c.1964 T > C (p.L655P) in this case and his father **(A)**. The patient’s mother and wife did not exhibit mutations at the same gene loci **(B)**.

The histopathologic findings of plantar skin lesion showed psoriasiform hyperplasia with compact hyperkeratosis, acanthosis, hypogranulosis, and parakeratosis of “corn-like” pattern in the stratum corneum with vesicular degeneration on the corneum and epidermis (Figures 3A,B). Next, we used whole-exome sequencing (including mitochondria) to analyze the patient’s family genes. *TRPV3* sequencing results revealed an identical heterozygous mutation c.1964 T > C (p.L655P) in the patient and his father (Figure 4A). Additionally, the mutation was not detected in the patient’s mother and wife (Figure 4B). This mutation is the 1964th base of the cDNA changes from T to C, resulting in the substitution of leucine for proline at amino acid position 655 (p.L655P). Based on the genetic findings, this familial case was consistent with an autosomal- dominant trait of inheritance.

**Figure 5:**
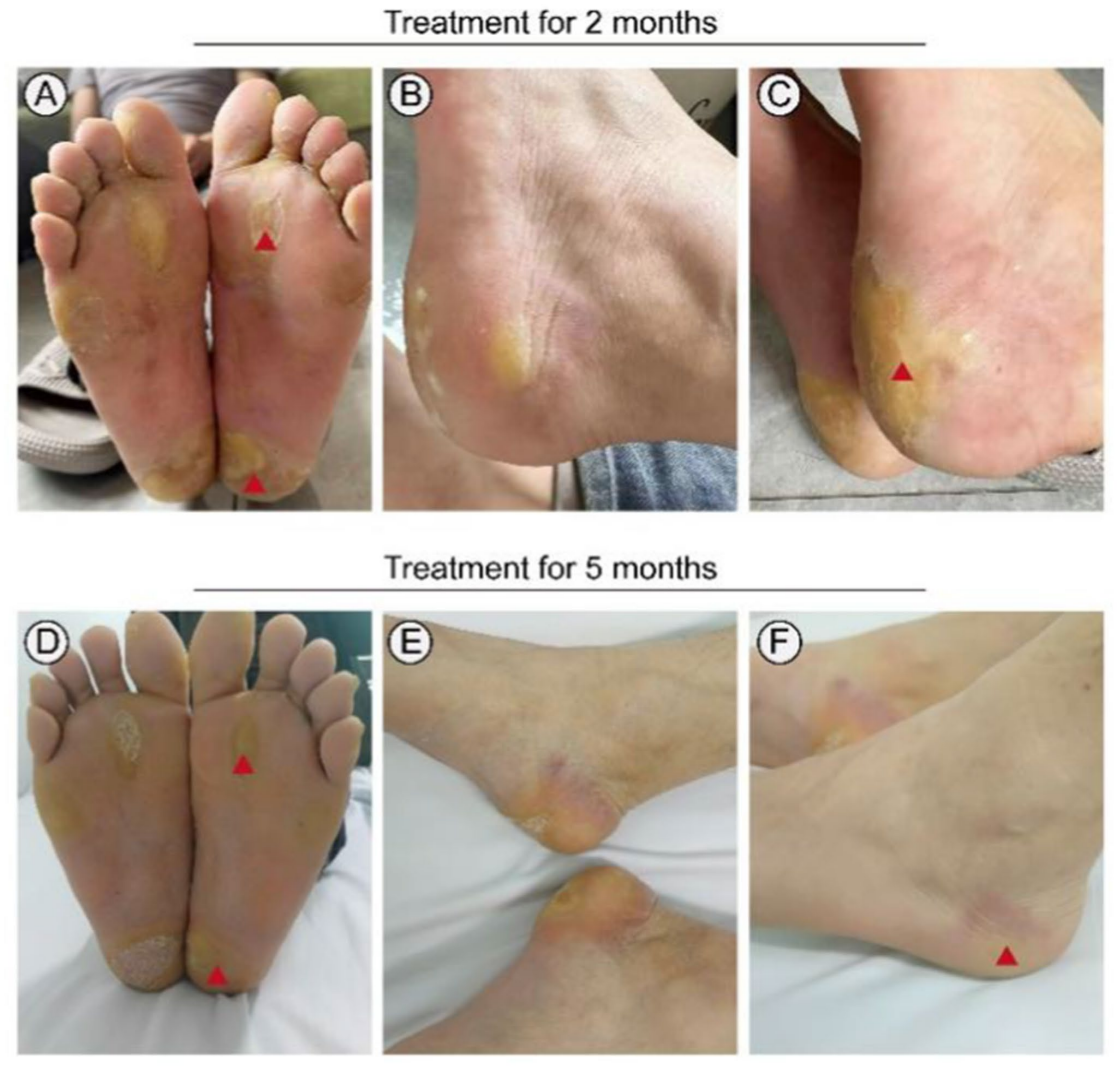
Treatment of this patient with erlotinib. Focal palmoplantar keratoderma and pseudoainhum were effectively relieved following therapy with erlotinib 75 mg for 3 months **(A–C)**. Plantar surface of both feet showing mild erythema and subtle hyperkeratosis following therapy with erlotinib 75 mg for 5 months **(D–F)**. Red triangle: Symptoms significantly improved.

Following informed consent and exclusion of contraindications, the patient received treatment with a combination of erlotinib and oral acitretin at a daily dose of 75 mg, considering his weight of 65 kg. During the treatment process, there were no significant severe side effects reported, only mild stomach discomfort and a slight burning sensation on the skin of the right forearm (Table 1). Within 3 months, significant improvement was noted, and subsequent examinations showed only mild redness and slight hyperkeratosis on both plantar surfaces after 5 months of treatment (Figure 5). The patient tolerated the medication effectively, with pain almost entirely alleviated and only intermittent flares. Notably, no severe adverse reactions were observed throughout the treatment course.

**Table 1:** The common adverse effect of erlotinib in the case.

Adverse effect	Severity	Description
Burning, tingling, numbness or pain in the skin	Mild	The patient reported a mild burning sensation on the skin of the right forearm on the first day after taking the medication.
Stomach discomfort, upset, or pain	Mild	After the first oral administration of the medication, the patient reported mild discomfort in the stomach.
Cough or hoarseness	None	
Difficult or labored breathing	None	
Fever or chills	None	
Lower back or side pain	None	
Painful or difficult urination	None	
Stabbing chest pain	None	
Tightness in the chest	None	
Severe chest pain	None	
Slurred speech	None	

## Discussion

In this study, we present a case report of a patient with *TRPV3* mutation-associated OS, who responded well to a novel use of erlotinib to treat pain and PPK. OS is an extremely rare hereditary skin disease, known for nearly a century, yet with fewer than a hundred reported cases, the prevalence remains unclear. The disease primarily manifests in the months following birth, with rare cases occurring in adulthood. OS is a genetically characterized by ulcerative palmoplantar keratosis, transgredient PPK resulting in flexural deformities and spontaneous amputation of the fingers or toes, usually accompanied by periorificial keratosis and hair loss (8). Notably, similar papules and plaques may appear over neck, axilla, cubital fossa, inguinal region and gluteal region (17). OS is observed in both sexes, although male cases are more frequent. Here we presented a typical familial case of OS with PPK, painful plaques on his feet, and cone-shaped hands. Similar OS symptoms were reported by Ning et al. (7). In addition to the sclerodactyly-like appearance and tapering fingers, pseudoainhum presented as a significant clinical feature in the current case. Literature suggests pseudoainhum may lead to damaging flexion contractures of the fingers (18). It is essential to consider Vohwinkel keratoderma, Huriez syndrome, and Mal de Meleda as differential diagnoses in cases of palmoplantar keratoderma. Pain and itching can vary in intensity, sometimes being severe. The patients in this study experienced intense pain in the weight-bearing area of the foot, significantly impacting their mobility and daily activities.

Most of reported OS cases are sporadic, with a few familial cases demonstrating various modes of inheritance (19, 20). *TRPV3* represents the first identified pathogenic gene for OS and is an autosomal dominant gene (6). *TRPV3* belongs to a large family of calcium-permeable transient receptor potential ion channel membrane proteins that contain six transmembrane domains (21). *TRPV3* is the primary identified causative gene linked to OS, inherited in an autosomal dominant pattern. To date, around 38 cases of OS linked to *TRPV3* mutations have been recorded, encompassing about 21 unique mutation sites. He et al. discovered that mutations in *TRPV3* disrupt the balance between keratinocyte proliferation and differentiation processes (22). Keratinocytes in the epidermis play a crucial role in forming the protective outer layer of the skin. Proper *TRPV3* function is vital for skin development. Mice lacking *TRPV3* exhibit thermal hypersensitivity, impaired skin barrier formation, and abnormal hair growth (23, 24). Recent findings of various mutations in human *TRPV3* associated with Olmsted Syndrome further emphasize the significance of *TRPV3* activity in the development of keratinocytes (14, 15, 25). The patient in this study possessed a mutation in the *TRPV3* gene identified as M1: c.1964 T > C: p.L655P. M1 indicates a heterozygous missense mutation, involving a change from thymine (T) to cytosine (C) at the 1964th position of the cDNA, resulting in the substitution of leucine with proline at the 655th codon. Sanger sequencing results validated the presence of the mutation in the index case, demonstrating a heterozygous genetic state.

Considering the presence of scleodactyly-like appearance and tapered fingers in his father, we speculate that it may be a special type of OS associated with *TRPV3* mutation. *TRPV3* has been associated with skin inflammation and wound healing, suggesting that abnormal activation of the *TRPV3* ion channel may contribute to the sclerodactyly-like appearance and tapered fingers phenotype (26, 27). Furthermore, *TRPV3* plays a vital role in itch and pain perception, regulating skin homeostasis, inflammation, and wound healing (21). Moqrich et al. found that mice without *TRPV3* struggle with sensing room temperature and responding to sudden heat, but their other senses are not affected (28). The patient in this study experienced recurring episodes of painful foot plaques, impeding walking and athletic activities due to the severity of the pain. Keratinocytes with *TRPV3* channels in the skin are not directly connected to sensory nerve endings. It’s suggested that certain small molecules may act as messengers between these cells and nerve endings, facilitating signal transmission. When *TRPV3*-positive skin cells are exposed to heat or *TRPV3* stimulants, they release substances like PGE2, ATP, nitric oxide, interleukin-1α, and transforming growth factor-*β* (29, 30). These substances sensitize and activate the nerve endings in the skin, completing signal transmission and causing specific effects. This process could lead to an accumulation of inflammatory factors at the site due to increased *TRPV3* expression, potentially contributing to peripheral neuropathy.

To elucidate the phenotypic spectrum of oral submucous fibrosis (OS), we conducted a comprehensive review of the clinical features associated with cases possessing known genetic underpinnings. Mutations in the *TRPV3* gene have been identified at seven distinct amino acid residues, specifically: p.Trp521, p.Gly568, p.Gly573, p.Gln580, p.Met672, p.Leu673, and p.Trp692, the majority of which are classified as autosomal-dominant missense mutations (6, 11, 31–33). Notably, the Gly573Cys, Gly573Ser, and Trp692Gly mutations exhibit gain-of-function properties and are associated with a pronounced clinical phenotype characterized by palmoplantar keratosis, periorificial keratotic plaques, alopecia, and symptoms such as warm-induced pain and itching (7). Overall, the disease severity displays substantial variability among patients harboring different *TRPV3* mutations, indicating a likely genotype–phenotype correlation linked to specific amino acid substitutions.

Mutations in the *TRPV3* gene can lead to diverse clinical presentations, resulting in significant variability among patients. In this case, both the patient and his father share the same mutation site, presenting with pronounced pain and localized severe keratinization on the sides of the metatarsals. However, the father also displays finger deformities, albeit milder compared to typical OS manifestations. Zhong et al. examined nine unrelated OS cases with differing clinical severities associated with *TRPV3* gene mutations to explore the relationship between genotype and phenotype (14).

The *TRPV3* channel interacts with and regulates epidermal growth factor receptor (EGFR) signaling. The *TRPV3* channel functions as a non-selective cation channel with a notable affinity for Ca^2+^. In response to various stimuli, it modulates the opening of Ca^2+^ ion channels within cells, thereby influencing cellular behavior. Studies have revealed that Olmsted Syndrome’s pathogenesis arises from acquired functional mutations in the *TRPV3* gene, causing heightened Ca^2+^ influx into keratinocytes. The increased calcium influx leads to the production of signaling molecules like TGF-*α* through “trans-activation.” This triggers EGFR phosphorylation, initiating the TGF-α/EGFR pathway that influences keratinocyte differentiation, causing cell death and severe skin keratinization (34). Erlotinib, a first-generation EGFR inhibitor, blocks the EGFR pathway by binding to the EGFR tyrosine kinase domain, preventing downstream signaling by ATP and the EGFR tyrosine kinase domain. In the present study, oral erlotinib was used to treat OS. After 3 months treatment of initiating erlotinib 75 mg, the PPK and pain were improved. Greco et al. have also shown that erlotinib improved pain and resolved PPK in OS patients with *TRPV3* mutations (15). Several case studies involving 12 patients, mostly children, demonstrated the effectiveness of oral erlotinib in OS treatment, highlighting its potential as a promising therapy (16, 35, 36).

This study is subject to certain limitations, such as the lack of a control group and a limited sample size. While the initial findings showed promise, larger-scale studies with extended follow-up durations are necessary to evaluate comprehensive clinical benefits, long-term safety profiles, disease progression post-treatment cessation, and responses in individuals with OS featuring diverse mutations. Furthermore, the absence of standardized assessment tools for OS posed a challenge in this investigation. Finally, this study did not conduct long-term follow-up on the patients and their families after treatment, particularly lacking data on the localized histological analysis of the skin following effective treatment.

In conclusion, we present a case study of a patient with OS dysfunction associated with a *TRPV3* mutation, specifically highlighting the positive outcomes observed with the administration of erlotinib for managing pain and PPK. Erlotinib, as a targeted therapeutic agent, demonstrated notable efficacy in alleviating the symptoms associated with these conditions. Additionally, we identified heterozygous frameshift mutation, c.1964 T > C (p.L655P), in exon 15 of the *TRPV3* gene, which was found in both the patient and his father. These findings suggest that targeted therapies like erlotinib may not only provide symptomatic relief but also address the underlying genetic factors contributing to this debilitating condition.

## Data Availability

The original contributions presented in the study are included in the article/supplementary material, further inquiries can be directed to the corresponding author/s.
